# Variable promoter methylation contributes to differential expression of key genes in human placenta-derived venous and arterial endothelial cells

**DOI:** 10.1186/1471-2164-14-475

**Published:** 2013-07-15

**Authors:** Jihoon E Joo, Ursula Hiden, Luciana Lassance, Lavinia Gordon, David J Martino, Gernot Desoye, Richard Saffery

**Affiliations:** 1 Cancer and Disease Epigenetics, Murdoch Childrens Research Institute, Royal Children’s Hospital, Flemington Road, Parkville, Melbourne, Australia; 2 Department of Obstetrics and Gynaecology, Medical University of Graz, Graz, Austria; 3 Bioinformatics Department, Murdoch Childrens Research Institute, Royal Children’s Hospital, Melbourne, Australia; 4 Department of Paediatrics, The University of Melbourne, Melbourne, Australia

**Keywords:** Endothelial cells, DNA methylation, Epigenetics, Placenta, NOS3, Gene expression, HPAEC, HPVEC, Reprogramming differentially methylated region

## Abstract

**Background:**

The endothelial compartment, comprising arterial, venous and lymphatic cell types, is established prenatally in association with rapid phenotypic and functional changes. The molecular mechanisms underpinning this process *in utero* have yet to be fully elucidated. The aim of this study was to investigate the potential for DNA methylation to act as a driver of the specific gene expression profiles of arterial and venous endothelial cells.

**Results:**

Placenta-derived venous and arterial endothelial cells were collected at birth prior to culturing. DNA methylation was measured at >450,000 CpG sites in parallel with expression measurements taken from 25,000 annotated genes. A consistent set of genomic loci was found to show coordinate differential methylation between the arterial and venous cell types. This included many loci previously not investigated in relation to endothelial function. An inverse relationship was observed between gene expression and promoter methylation levels for a limited subset of genes implicated in endothelial function, including *NOS3*, encoding endothelial Nitric Oxide Synthase.

**Conclusion:**

Endothelial cells derived from the placental vasculature at birth contain widespread methylation of key regulatory genes. These are candidates involved in the specification of different endothelial cell types and represent potential target genes for environmentally mediated epigenetic disruption *in utero* in association with cardiovascular disease risk later in life.

## Background

Mounting evidence linking environmental exposures in early life to later risk of cardiovascular disease has led to intense interest in the process of vasculature development *in utero*[1-3]. Primarily, arteries and veins are defined by physiologic factors such as the direction and pressure of blood flow and by functional and anatomical differences such as the arrangement of smooth muscle cells around the vessels. In general, arteries carry oxygenated blood, and have tighter endothelial junctions, whilst veins carry deoxygenated blood and have looser endothelial junctions [4]. The feto-placental vascular system differs from that of most other human organs, because the arteries carry the deoxygenated blood coming from the fetus whilst the veins carry the oxygen enriched blood.

Not surprisingly, given their different physiologic functions, the identity of arterial and venous endothelial cells is established before the onset of circulation [5-7] in association with distinct gene expression signatures that define endothelial cell identity [8-10]. This includes endothelial nitric oxide synthase (eNOS) encoded by *NOS3*, von Willebrand factor (vWF), vascular endothelial cadherin (VE-cadherin, *CDH5*), intracellular adhesion molecular-2 (*ICAM-2*), endothelial growth factor receptor tyrosine kinases VEGF-R1 (*Flt-1*) and VEGF-R2 (*Flk-1, KDR*), angiopoietin receptors Tie-1 and Tie2 (*TEK*) and NOTCH4 [Reviewed in [11,12]]. In addition, a number of transcription factors have been shown to be preferentially expressed in endothelial progenitor cells and mature endothelial cells and have been argued to orchestrate the expression of such genes [11]. Despite this, the associated epigenetic regulators determining this specific gene expression profile are largely unclear.

Numerous lines of evidence suggest that the endothelial compartment represents a potential target tissue for transmission of environmentally mediated risk of complex disease, potentially via a process of epigenetic disruption in early development [2,13]. Epigenetic mechanisms, including DNA methylation, are now widely accepted to underpin developmentally regulated changes in cell morphology and function [14], however little is known about the role of such modifications, and their relative plasticity, during endothelial cell development. It is clear that one of the key regulators of vascular function, endothelial nitric oxide synthase (eNOS) is under epigenetic control by several mechanisms, including DNA methylation [15]. Indeed the chromatin structure of the eNOS promoter is transcriptionally permissive only in cells of the endothelial compartment, such as those isolated from human umbilical vein, mouse aortic, and pooled human dermal microvascular endothelial cells [15,16]. Altered chromatin has also been reported for other endothelial-specific genes including *vWF*[17], *NOTCH4*[18]. Furthermore, studies have shown changes in epigenetic marks induced by oxidative stress (i.e. hypoxia) in *VEGF*[19] and *NOS3*[20], supporting the potential epigenetic roles in tissue-specific regulation of those genes. Recent genome-scale comparison of DNA methylation in dermal derived lymphatic and blood-derived endothelial cells has highlighted the role of differential methylation in the specification of endothelial function [21], but beyond these limited data, little is known about the relationship between epigenetic modifications and gene expression underpinning endothelial phenotype or developmental stage at the genome-wide level. As a first step towards addressing these questions, we have examined the relationship between genome-scale DNA methylation and gene expression in purified human placental arterial and venous endothelial cells (HPAEC and HPVEC).

## Results and Discussion

Placenta-derived venous and arterial endothelial cells play especially important functional roles (e.g. nutrition, cholesterol delivery) as part of the fetal-maternal supply line [22,23]. Furthermore, several physiological differences exist between these two cell types [24] and they differ in their degree of maturity. Whereas placental arterial endothelial cells have a mature arterial phenotype (fully differentiated), venous derived cells show a juvenile (less differentiated) phenotype, potentially representing a pool of endothelial progenitor cells [9].

We compared DNA methylation in 9 purified cell populations each of Human Placental Arterial Endothelial Cells (HPAEC) and venous equivalents (HPVEC). After removing probes that did not pass a quality control cutoff (i.e. probes with >0.05 p-value detection) and probes on sex chromosomes, a total of 351,952 probes remained that were common to all datasets for inclusion in subsequent analyses. β-values (DNA methylation values between 0 and 1, approximating 0-100% methylation) were calculated for each probe in HPAEC and HPVEC samples.

### General hypomethylation in placental venous relative to arterial endothelial cells

**Figure 1:**
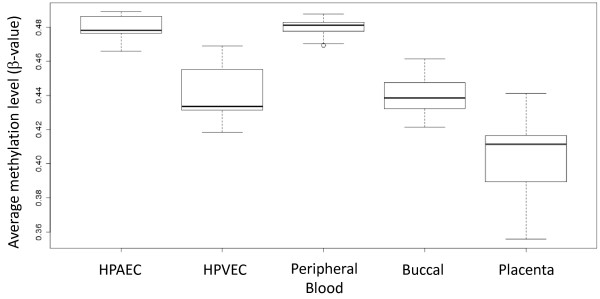
**Comparison of average DNA methylation levels across >300,000 CpG sites in HPAEC (n=9) and HPVEC (n=9).** The average β-value across the entire dataset was generated as a proxy for global methylation levels as previously described [25]. Hypomethylation of HPVEC was observed (average β = 0.434) relative to HPAEC (average β = 0.479). Peripheral blood (n=40), Buccal (n=59) and Placenta (n=19) were included for comparative purposes.

As a first comparison of methylation within the two cell types, we calculated an average β-value across the entire dataset as a proxy for global methylation levels in venous and arterial cell samples as previously described [25]. Interestingly, we found clear evidence for a general hypomethylation in HPVEC (average β = 0.434) relative to HPAEC (average β = 0.479), with the latter comparable to peripheral blood average methylation, whereas HPVEC levels were more similar to that of buccal cells taken at birth. Both HPAEC and HPVEC were generally more methylated on average than placental tissue (Figure 1).

### Widespread epigenetic differences in placental arterial and venous endothelial cell compartments

**Figure 2:**
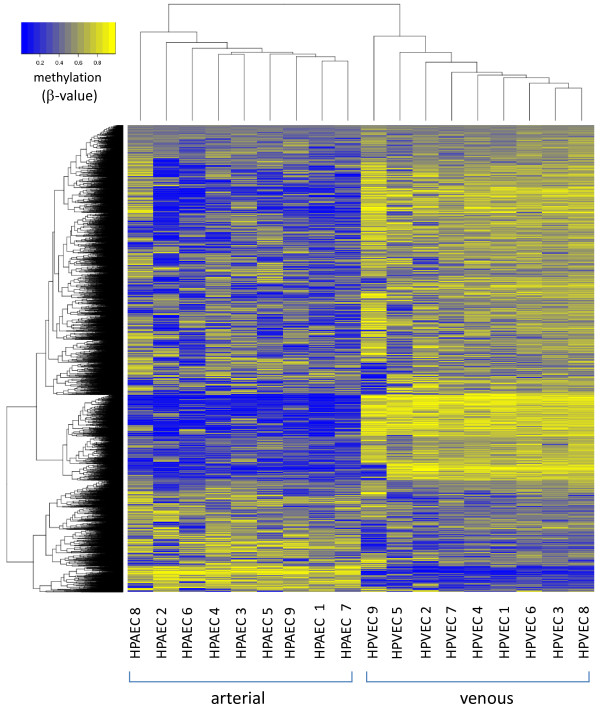
**Unsupervised hierarchical clustering (dendrogram) and heatmap of 1000 most highly variable probes in HPAEC and HPVEC samples.** Despite evidence of variability in DNA methylation profile within each cell type, HPAEC and HPVEC samples are clearly discriminated according to methylation profile.

Importantly, where tested, HM450 DNA methylation levels showed strong correlations with those derived using locus-specific SEQUENOM MassARRAY EpiTYPER platform (Additional file 1: Figure S3), validating the HM450 platform, in accordance with other recent studies [26-29]. Unsupervised hierarchical clustering of variable probes in this dataset (SD/mean > 0.4; 151453 probes) clearly discriminated arterial from venous samples (Additional file 1: Figure S1) highlighting the genome-wide DNA methylation differences between these endothelial compartments. HPAEC were more variable as a group than HPVEC in terms of DNA methylation profile. This discriminatory capacity was maintained following unsupervised clustering of the 1000 most highly variable probes (Figure 2). Further, a similar comparison with recently reported methylation datasets generated from cultured adult dermal blood and lymphatic endothelial cell populations [21] with our dataset, along with unpublished Human Umbilical Vein Endothelial cell (HUVEC) data, highlights the distinct DNA methylation profile of endothelial source from different vascular compartments. HPVEC show the most unique methylation profile, clustering separately from lymphatic and blood derived endothelial cells, HUVECs and HPAEC cells (Additional file 1: Figure S2).

To identify sets of differentially methylated probes (DMPs) between HPAEC and HPVEC, average β-values were calculated for each probe in HPAEC and HPVEC groups. Linear regression analysis revealed a large set of significantly differentially methylated probes (95,266 in total; Benjamini-Hochberg adjusted p-value ≤ 0.05). A total of 15,115 probes, associated with 5,142 genes, showed elevated methylation in HPVEC relative to HPAEC (average β-value difference ≥0.10; Additional file 2: Table S2) whereas 53,725 probes associated with 12,659 genes showed the reciprocal pattern (Additional file 3: Table S3). These data highlight the coordinated nature of methylation change, with blocks of probes (differentially methylated regions – DMRs), showing a common shift in the direction and magnitude of methylation change between cell types.

In order to examine the potential for altered DNA methyltransferase activity to explain the observed global DNA methylation differences described above, we extracted methylation and gene expression data for found evidence for differential methylation of both the DNMT1 and DNMT3A (but not DNMT3B) genes, with elevated average methylation in both promoter regions in HPVEC relative to HPAEC (Additional file 4: Table S1). Further, this was associated with a reciprocal pattern of gene expression specifically for DNMT3A consistent with methylation induced down regulation of DNMT activity in the placental venous compartment (Additional file 4: Table S1).

Of particular interest to endothelial cell biology, many DMP/DMRs were identified in genes previously implicated in endothelial functioning, including nitric oxide synthase 2 (iNOS) [12,30] and *NOS3* (encoding eNOS) [15,31], von Willebrand Factor (*VWF*) [11], *NOTCH4*[18], *VEGFA, VEGFC*[32]*,SELE*[33], *FLT*[34]*,* and *KDR*[35] genes, although the number of DMPs and direction of methylation difference between cell types was variable (Additional file 2: Tables S2 and Additional file 3: Table S3).

**Figure 3:**
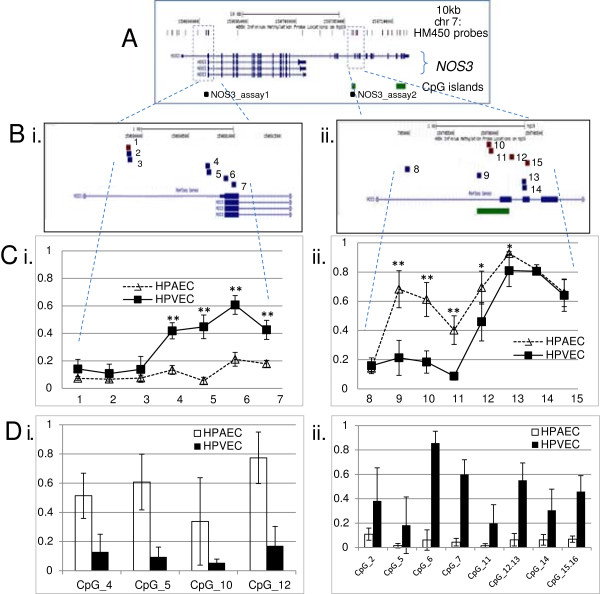
***NOS3 *gene methylation levels vary according to genomic location in HPAEC and HPVEC cells. A**. UCSC Genome Browser View of *NOS3* showing chromosomal coordinates (Chr7 - chromosome 7), location of HM450 array probes, CpG sites (black vertical dashes), alternative splice variants including differential exons (blue bar) and introns (arrowed lines), CpG islands (green bar). **B**. Magnified view of regions of differential methylation of the *NOS3* gene between HPAEC and HPVEC. **C**. Graphical summary of mean *NOS3* methylation level (y-axis) in HPAEC (open triangles; n=9) relative to HPVEC (closed squares; n=9) for HM450 probes 1–14 in A. (x-axis) highlights a region of methylation of the major *NOS3* promoter (i) and reciprocal hypomethylation of a downstream CpG island (iii) in HPVEC relative to HPAEC cells . Error bars denote 95% confidence interval and asterisks denote level of significance of array data according to adjusted p-value from linear regression analysis. **D**. DNA Methylation patterns detected by Sequenom EpiTYPER around the two regions corresponding to the HM450 probes. This cross-platform analysis further confirms the methylation of the major *NOS3* promoter in HPVEC (i) and the reciprocal hypomethylation of the downstream CpG island (ii) Error bars – 95% confidence interval.

Interestingly, many genes showed a reciprocal effect on methylation profile according to genomic location relative to the transcription start site. For example, *NOS3*, showed two regions of highly significant differential methylation. The first, located in the major promoter previously described to drive endothelial expression, encompassed 4 probes with higher mean methylation in HPVEC relative to HPAEC cells, while a reciprocal mean methylation pattern was observed for 5 probes in a downstream exon-associated region in a previously described, placenta-specific variant of this gene (Figure 3). Locus-specific analysis of methylation confirmed highly differential methylation across each of these regions (Figure 3D), however the regulatory function (if any) of this second region remains to be determined. Such complex patterns of methylation within individual genes have recently emerged as a common feature in gene regulation in humans [36-38].

*NOS3* is constitutively expressed in the vascular endothelium and plays a critical role in cardiovascular physiology as evidenced by systemic and pulmonary hypertension, abnormal vasculature, defective angiogenesis, poor healing in response to injury and impaired mobilization of stem cells, in eNOS-null mice [reviewed in [39]]. Disruption of methylation in either of these regions early in development could be a modifiable risk factor for disease in later life.

### Complex interplay between endothelial DNA methylation and gene expression

**Figure 4:**
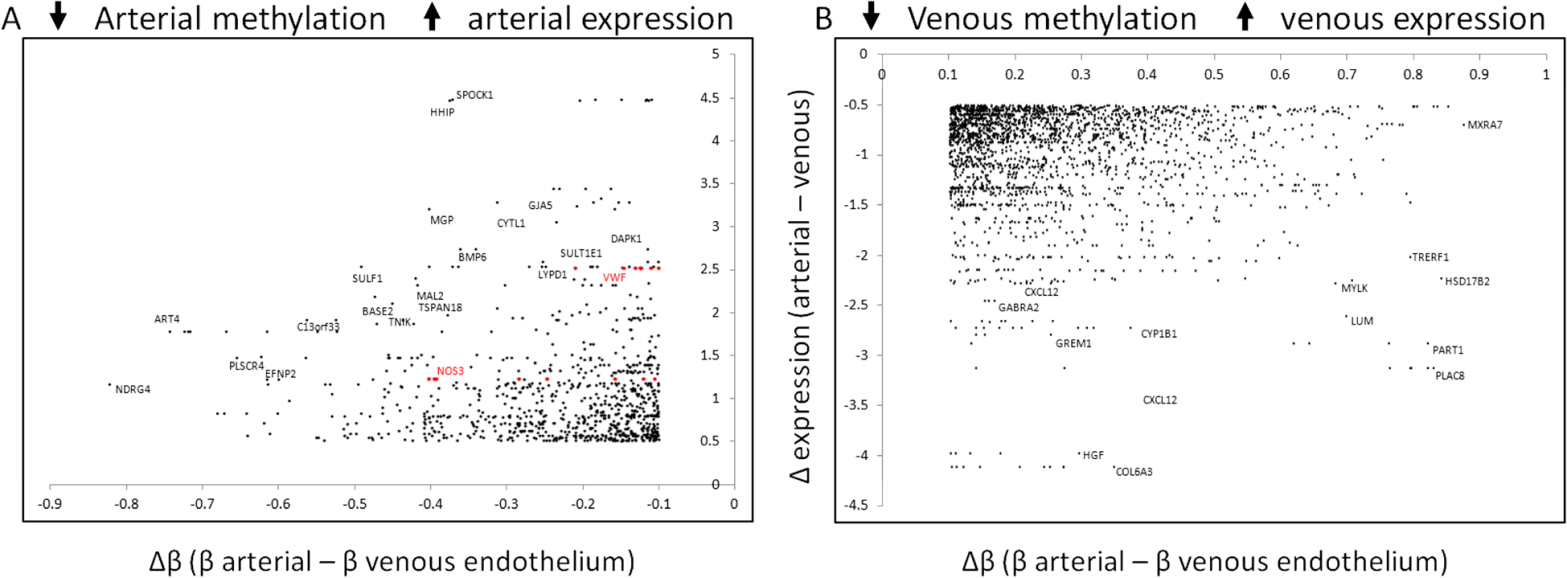
**Scatterplot of DNA methylation (x-axis) and gene expression (y-axis) differences between HPVEC and HPAEC for genes showing reciprocal methylation/gene expression change.** Gates were set at the limits of technical noise (methylation +/− 10%, gene expression +/− 0.5 fold change). Each set of HM450 probe datapoints (x-axis) with a common set of gene expression values (y-axis) represents a gene likely to be under epigenetic regulation by DNA methylation with decreased methylation associated with elevated gene expression in HPAEC relative to HPVEC **(A)** and vice versa **(B)**. Genes highlighted in red included NOS3 and vWF. Note that in many instances, genes contain multiple HM450 probes for each gene expression measure.

Extensive epigenetic studies have highlighted the complex relationship between DNA methylation and gene expression levels. In addition to the well documented role of elevated methylation at promoter-associated CpG islands in down-regulation of gene expression, it is also apparent that some methylation marks at these (and other) sites may be independent of gene expression status, or even predict active expression, as observed in several DMRs of imprinted genes or in the case of gene-body methylation [36-38]. Thus, in order to examine the relationship between DNA methylation and gene expression, we integrated the methylation and gene expression data sets. For each HM450 probe, differences in β-values between the two groups (Δβ) were calculated and these were compared with corresponding gene expression differences (Additional file 1: Figure S5). By setting cutoffs in average methylation difference higher than 10% (Δβ > 0.1) and log2 fold change in gene expression of ~1.4 (0.5 logFC), we identified a subset of genes that showed a reciprocal relationship between methylation and expression level in HPVEC and HPAEC (Figure 4; Additional file 1: red dots in Figure S5), consistent with a role of increasing methylation in down-regulation of gene expression. This includes 866 HM450 probes showing lower methylation in association with elevated expression of 306 genes in arterial cells (Additional file 5: Table S6), and 2388 probes covering 513 genes showing the same relationship in venous cells (Additional file 5: Table S7).

In order to test for any pattern regarding genomic location and likelihood of regulating gene expression, we examined the proportion of each probe type showing coordinate changes in differential methylation and gene expression, according to annotation (promoter-associated, location within 1500 bp of transcription start site, gene-body, and enhancer-associated). This revealed that the majority of probes were located in enhancer or upstream regulatory (TSS1500, promoter) regions (Table 1, Additional file 1: Figure S6).

**Table 1:** Number of differentially methylated probes sorted by genomic region

	**All probes**		**Probes located within 1500 bp upstream, 5'UTR and 1st Exon (“non‒gene body” probes)**			**5'UTR and 1st Exon (“non‒gene body” probes) Transcription Start Site (Excludes 5’UTR/1st Exon)**		
Quadrant number Additional file 1: Figure S4)	Probe Count	Proportion to total probes in group	Probe Count	Proportion to total probes in group	Proportion to all analysable probes	Probe Count	Proportion to total probes in group	Proportion to all analysable probes
	351952	100.00%	184099	100.00%	52.31%	130656	100.00%	37.12%
**Q1**	866	0.25%	462	0.35%	0.13%	292	0.22%	0.08%
**Q2**	3441	0.98%	955	0.73%	0.27%	619	0.47%	0.18%
**Q3**	2388	0.68%	1011	0.77%	0.29%	627	0.48%	0.18%
**Q4**	711	0.20%	301	0.23%	0.09%	179	0.14%	0.05%
	**Probes on Enhancer Regions**		**Promoter Associated Probes**			**Gene Body Only (not 5’UTR or 1st Exon)**		
Probe Count	Proportion to total probes in group	Proportion to all analysable probes	Probe Count	Proportion to total probes in group	Proportion to all analysable probes	Probe Count	Proportion to total probes in group	Proportion to all analysable probes
59317	100.00%	16.85%	91601	100.00%	26.03%	146423	100.00%	41.60%
275	0.46%	0.08%	97	0.11%	0.03%	359	0.25%	0.10%
1239	2.09%	0.35%	145	0.16%	0.04%	2220	1.52%	0.63%
960	1.62%	0.27%	214	0.23%	0.06%	1221	0.83%	0.63%
286	0.48%	0.08%	65	0.07%	0.02%	379	0.26%	0.11%

Q1, probes showing less methylation (<-10%) and higher expression (>0.5 LogFC); Q2, probes showing higher methylation (>10%) and expression; Q3, probes showing higher methylation and less expression (<-0.5 LogFC); Q4, probes showing less methylation and expression in HPAEC relative to HPVEC, Genomic regions may overlap between one or more categories (e.g. a probe can be located in 1500 bp from upstream and also be associated with Enhancer and/or Promoter).

Interestingly, endothelial genes likely to be directly regulated by DNA methylation in our dataset included *NOS3, vWF, APOLD1, ANGPTL2* genes. Many endothelial genes show both differential methylation at upstream regulatory regions and gene expression differences, but not always in the anticipated reciprocal direction (Table 2A, Additional file 5: Table S8; Additional file 1: Figure S5).

**Table 2:** DNA Methylation of probes located near transcription start sites of selected genes associated in endothelial function and corresponding gene expression levels

**Selected genes showing changes in both promoter methylation and gene expression**				
**Gene symbol**	**Description**	**Gene expression LogFC (HPAEC ‒ HPVEC**	**Average β of HPAEC ‒HPVEC**	Probes located in promoter region / total number of probes*
*NOS3*	Nitrix Oxide Synthase	-1.233	0.314	6/28 (21%)
*vWF*	von Wildebrand Factor	-2.523	0.124	4/38 (11%)
*MGP*	Matrix Gla protein	-3.202	0.225	1/3 (33%)
*GJA5*	Connexin40	-3.434	0.122	7/15 (47%)
*APOLD1†*	apolipoprotein L domain containing 1 [Homo sapiens]	-0.061	0.684	7/32 (22%)
*HIF3A†*	Hypoxia Inducible Factor 3, alpha subunit	-0.599	-0.161	8/23 (35%)
*EphB1†*	Ephrine type-B receptor 1	1.050	0.284	2/36 (6%)
*ANGPTL†*	Angiopoietin-like 2	0.768	-0.178	2/6 (33%)
*SELE†*	E-Selectin	0.934	0.165	2/3 (67%)
*FLT1 (VEGFR1) †*	Vascular Endothelial Growth Factor Receptor 1	0.930	0.333	3/28 (11%)
*COL6A3*	Collagen, type VI, alpha 3	4.117	-0.128	9/53 (17%)
*HSD11B1*	Hydroxysteroid (11-beta) Dehydrogenase 1	1.092	-0.177	8/9 (89%)
*VEGFC*	Vascular Endothelial Growth Factor C	0.662	-0.101	5/13 (38%)
*HGF*	Hepatocyte Growth Factor (hepapoietin A; scatter factor)	3.973	-0.092	7/10 (70%)
*ANGPT1*	Angiopoietin 1	1.172	-0.210	2/16 (13%)
**Genes showing expression changes but no changes in DNA methylation**				
**Gene symbol**	**Description**	**Gene expression LogFC (HPAEC ‒ HPVEC**	**Average β of HPAEC ‒HPVEC**	Probes located in promoter region / total number of probes*
*ARG2*	Arginine 2	0.445	<0.001	11/15 (73%)
*DLL1*	Delta like protein 1 precursor	0.077	0.005	12/38 (32%)
*JAG1*	Jagged 1 precursor	0.605	<0.001	10/24 (42%)
*JAG2*	Jagged 2 precursor	0.369	<0.001	8/47 (17%)
*KDR (VEGFR2)*	Kinase Insert Domain Receptor	1.552	0.003	9/16 (56%)
*HEY2*	hairy/enhancer‒of‒split related with YRPW motif 2	1.506	<0.001	11/21 (52%)
*NOTCH*	neurogenic locus notch homolog protein 4 preproprotein	0.429	0.024	3/160 (2%)
*EphB2*	neurogenic locus notch homolog protein 4 preproprotein	0.262	0.007	5/46 (11%)
*NRP1*	Neuropilin 1	0.529	0.002	6/29 (21%)
*EphB4*	Ephrine type‒B receptor 4	0.732	<0.001	7/29 (24%)
*EphB4*	Intercelluar adhesion molecule‒2	0.808	0.029	11/15 (73%)

* denotes number of probes located near promoter regions of each gene where highly differential DNA methylation patterns were detected between two cell types out of total numbers of probes associated with each gene. † denotes probes where strong DNA methylation differences and gene expression patterns were detected but not inverse relationship. It may indicate there exists other regulatory regions or gene expression is regulated by epigenetic mechanism other than DNA methylation.

The *FLT1* and *KDR* genes code for two different subtypes of VEGF receptors. These genes have previously been shown to be expressed in most endothelial cells including aortic, vein, microvessel and in many tumors, albeit variably across tumors and individuals [40-42]. FLT1 and KDR are postulated to play a significant role in angiogenesis and/or tumor progression by influencing the activity of *VEGF*[43,44]. These genes are shown to be hypermethylated in some cancer cell lines (e.g. colon, stomach) [41]. In our study, *FLT1* showed differential promoter methylation and gene expression in HPAEC and HPVEC, however expression and methylation were positively correlated rather than anti-correlated (Table 2). In contrast, the *KDR* gene was differentially expressed between two cell types but this was independent of any DNA methylation differences in the vicinity of the transcription start site. These findings are interesting given that previous studies have shown down regulation of each of these genes in association with promoter methylation in various cell lines, while cell lines expressing KDR (including HUVECs) appear universally unmethylated [41,45].

The *TLR2* gene has previously been shown to be expressed in endothelial cells in a cell-type specific manner [46]. We found no significant gene expression difference in *TLR2* in HPVEC relative to HPAEC (average log FC < 0.073) despite significant differences in promoter methylation levels, with HPVEC having higher regional methylation (average Δβ = 0.213), spanning seven HM450 probes, located proximal to the transcription start site. The activity of this gene in HUVECs has previously been shown to be regulated by inflammation in the absence of altered DNA methylation in HUVECs [47].

In combination these data highlight the differential and complex role of epigenetics in the regulation of gene expression in endothelial cells of different origin, with several genes likely to be regulated by alternative epigenetic mechanisms (such as histone modification and/or non-coding RNAs) in a cell context-dependent manner. Differential utilization of genomic regulatory elements or expression of upstream transcription factors may also contribute to the observed pattern of expression.

### Epigenetic regulation of miR expression in endothelial cells?

Multiple examples of miRNA gene differential methylation were apparent in HPAEC and HPVEC cells (Additional file 5: Table S9), including miRs previously implicated in regulation of endothelial function and/or angiogenesis such as miR-126 and miR-130A [48]. Interestingly miR-200 family members (miR200-A, –B and –C), involved in regulation of VEGF signaling in endothelial cells [49], were found to be more highly methylated in HPAEC than HPVEC. Furthermore, miR-10A-associated probes, previously linked with arthero-function, were specifically hypermethylated in HPAEC relative to HPVEC [50]. In contrast, the gene encoding miR-125B, a translational suppressor of VE-cadherin [51], were hypermethylated in HPVEC, relative to HPAEC. Despite the clear evidence of differential methylation, an examination of miR expression profiles failed to reveal a corresponding difference in gene expression between the two cell types (data not shown). Thus, the biological significance of the methylation differences remains.

### Ontology of genes associated with DMRs

In order to more fully understand any biological/cellular functions subject to coordinated regulation in endothelial cells, we performed gene ontology and Ingenuity Pathway Analyses (http://www.ingenuity.com) on differentially methylated genes. Prior to the analysis, the list of DMPs was selectively culled to focus on those probes predicted to play a role in gene regulation according to genomic location (associated with gene promoters, enhancers, 5’ UTR, or TSS associated regions as specified by the HM450 manifest annotation file HM450_V1.2). Ingenuity Pathway Analysis revealed an enrichment of genes involved in biological functions such as “Tissue Development”, “Cardiovascular System Development and Function”, and “Tissue Morphology” as differentially methylated in HPAEC and HPVEC (Additional file 5: Table S4). Many of these genes warrant further investigation in the development of arterial or venous endothelial cell phenotype. Gene Ontology Analysis performed by DAVID (http://david.abcc.ncifcrf.gov/, [52]) highlighted related biological processes associated with the “Extracellular matrix”, previously shown to be critical for proper vascular development [53], and “Plasma membrane” and “Vasculature Development” (Additional file 5: Table S5). Importantly, coordinated regulation of genes involved in specific cellular pathways such as “cardiovascular pathway genes” was apparent (Additional file 1: Figure S4).

## Conclusion

Placenta-derived arterial and venous endothelial cells differ in both their functional characteristics [24] and differentiation state [9]. Whereas arterial cells are mature and fully differentiated, their venous counterparts have been regarded as immature, representing a juvenile phenotype with a high degree of plasticity. We speculate that differential phenotype of these cells is largely driven by distinct gene expression changes, many of which are mediated by promoter methylation differences in key genes identified here. At present it is not possible to distinguish which epigenetic changes are associated with the degree of maturity of the cells, and which drive more general distinct differences in venous vs arterial characteristics. This will require further investigation.

The two cell types examined show a distinct difference in global DNA methylation level, with the HPVEC hypomethylated relative to HPAEC, and other endothelial cells from a variety of tissues. The low average methylation seen in the venous compartment is reminiscent of the hypomethylation seen in placental tissue and may be one of the underlying mechanisms by which environmental cues modulate their phenotype to adapt to the microenvironment. Indeed, the HPVECs are more sensitive to changes in their local environment *in vitro* relative to the HPAEC counterparts [53].

To the best of our knowledge this is the first study comparing global gene expression and DNA methylation of primary arterial and venous endothelial cells isolated from the same organ. In general our data have revealed a highly coordinated series of DNA methylation events, many of which are directly implicated in regulating underlying gene expression levels, while the functional significance of others is less apparent. Included in our dataset are numerous genes previously not studied in relation to endothelial function, that warrant such an investigation in future studies. The combination of DNA methylation and gene expression profiling of early life endothelial cells represents a powerful approach to identify candidate loci potentially subject to environmentally mediated epigenetic disruption, in association with modified risk of later complex diseases involving cardiovascular dysfunction.

## Methods

### HPAEC and HPVEC: isolation and culturing

Primary HPAEC and HPVEC were isolated from arteries and veins, respectively dissected from placentas after uncomplicated vaginal delivery as described previously [9]. Each of the 9 venous and arterial endothelial cell pairs used here was isolated from the same vascular loop of 9 full term human placentas. Cells were cultured on 1% (v/v) gelatin-coated plates using Endothelial Basal Medium (EBM, Cambrex, Clonetics™, Walkersville, MD) supplemented with the EGM™-MV BulletKit (Clonetics™). They were characterized by internalization of acetylated low-density-lipoprotein and immunohistochemical staining for the endothelial cell marker von Willebrand factor and negative staining for fibroblast-specific antigen and smooth muscle actin [9]. Only HPAEC and HPVEC pairs, i.e. isolated from the same placentas, were used in order to minimize variance.

### Nucleic acid purification and QC

Total RNA from HPAEC and HPVEC was isolated with RNeasy mini kit (QIAGEN, Dusseldorf, Germany) and scrutinized for quality on the BioAnalyzer BA2100 (Agilent, Santa Clara, CA, USA) with the RNA 6000 Nano Chip Kit (Agilent, Cat No 5067–1511). The RIN (RNA Integrity Number) of the samples ranged between 8.7 and 10. Genomic DNA from HPAEC and HPVEC was isolated using phenol/chloroform density gradient centrifugation method as described previously [54].

### Genome-scale DNA methylation analysis: data acquisition and processing

A total of 1 μg of DNA isolated from 9 HPAEC and 9 HPVEC cell populations was bisulphite converted using the MethylEasy™ bisulphite modification kit (Human Genetic Signatures, Sydney, Australia), according to the manufacturer’s instructions. Unpublished HUVEC data used for unsupervised hierarchical clustering were obtained from purified cells isolated as previously described [55]. Unpublished HM450 data from buccal cells, peripheral blood and placental tissue, used for a comparative analysis of average β values across over 300,000 genomic loci, was kindly provided by Drs Jeff Craig and Boris Novakovic, Murdoch Childrens Research Institute. Conversion efficiency was assessed by bisulphite-specific PCR. Hybridization of bisulphite-treated samples to Illumina Infinium Human Methylation450 (HM450) Beadchips was performed at the Australian Genome Research Facility (AGRF). Raw data files were exported from Genome Studio (Illumina, San Diego, CA) into the R statistical environment (http://cran.r-project.org/index.html). Infinium HM450 data was normalised using the SWAN method from the *minfi* package available from Bioconductor [56,57]. This has been specifically designed for such platform where a bias from the two types of probes is apparent. M-values were calculated after removing probes on the sex chromosomes to eliminate any potential gender bias and any poor performing probes, defined as those with a detection p-value cut-off > 0.05 in any sample. β-values were derived from intensities as defined by the ratio of methylated to unmethylated probes given by β =M / (U+M) and were used as a measure of effect size.

### Gene expression array analysis: data acquisition and processing

Total RNA was labeled using Affymetrix GeneChip® Whole Transcript (WT) Sense Target Labeling Kit (Affymetrix, Santa Clara, CA, USA; Cat No. 900652) and then prepared for hybridization. For expression analysis RNA was hybridized against GeneChip® Human 1.0 ST arrays (Affymetrix, Cat No. 901087) according to the manufacturer’s instructions. Labeling and hybridization controls were evaluated with Expression Console EC 1.1. Hybridizations and analysis were carried out at the Division Core Facility for Molecular Biology at the Centre of Medical Research at the Medical University of Graz. Microarray data were analysed with Partek Genomic Suite v6.4 software (Partek Inc, St Louis, MO, USA). The import process of the CEL files contained RMA normalization (robust multi-chip average) including background correction, quantile normalization across all arrays and median polished summarization based on log transformed expression values. Significant different genes were extracted with FDR5% / p<0.005 / p<0.05 using ANOVA. Annotations were obtained from NetAffx (Affymetrix).

### Statistical analysis and bioinformatics

The Benjamini–Hotchberg method was used for controlling the false discovery rate and correct for multiple testing when comparing HPAEC and HPVEC methylation [58]. The HM450 data (M-value) underwent unsupervised hierarchical clustering analysis using the *lumi* package [59]. Linear regression analysis was performed using the *limma* package [60]. For combined gene expression and DNA methylation analysis, Δβ values (average β-values of HPVEC subtracted from average values of HPAEC) were plotted against the average Log_2_ fold gene expression change of HM450 gene-associated probes. To determine the association between genomic location and differential methylation status, HM450 probes were exclusively assigned to one of the following groups based on the HM450 manifest annotation file version 1.2: promoter associated; enhancer associated; within 1500 bp upstream of a transcription start site (TSS); within 1500 bp upstream of a gene, 5’UTR and first exon; and probes located in gene body and 3’UTR only. Gene Ontology and pathway analysis was performed using the DAVID bioinformatics tool (david.abcc.ncifcrf.gov/) [61] and Ingenuity Pathway Analysis (http://www.ingenuity.com) under the default settings.

### Gene specific DNA methylation

*NOS3* locus-specific methylation was performed using the Sequenom EpiTYPER MassARRAY platform (Sequenom, San Diego, USA) as previously described [62,63]. Amplicons were designed using EpiDesigner software (http://www.epidesigner.com/) and amplification conditions were as follows: 95°C for 10 min; 95°C for 10 s, 56°C for 30 s, and 72°C for 1 min 30 s for 40 cycles; 72°C for 7 min. Primer and target sequences, along with amplification cleavage product patterns and analyzable CpG units are provided in Additional file 1: Figure S7.

### Ethics declaration

Human placental tissue was obtained at term of gestation from uncomplicated pregnancies. All women were lean (BMI 20–24.9), non-smokers and with blood pressure in the normal range. All underwent an oral Glucose Tolerance Test between weeks 24 and 28 and their blood pressure was measured at each visit. Values were in the normal range (ie. did not exceed threshold levels to classify the women as being diabetic/gestational diabetic or having pregnancy-induced hypertension/pre-eclampsia). Informed consent was obtained and ethical approval was granted by the ethics committee of the Medical University of Graz. This study meets the principles of the Declaration of Helsinki.

## Abbreviations

β (values): Beta (values), relative DNA methylation values; Δβ: Delta Beta, difference in Beta values; DMR: Differentially methylated region; DMP: Differentially methylated probe; HPAEC: Human placental arterial endothelial cells; HPVEC: Human placental venous endothelial cells; HUVEC: Human umbilical vein endothelial cells; RIN: RNA integrity number; TSS: Transcription start site.

## Competing interest

The authors declare that they have no competing interests.

## Authors’ contribution

RS and GD conceived the idea and designed the study. JEJ performed the DNA methylation experiments and the analysis. UH, LL and GD performed the isolation of endothelial cells and the gene expression experiments. LG and DM contributed to the bioinformatics analysis. JEJ and RS wrote the majority of the manuscript. All authors read and contributed to the final manuscript.

## Supplementary Material

Additional file 1: Figure S1 Unsupervised hierarchical clustering of probes showing variable DNA methylation levels (coefficient of variation >0.4) in HPAEC and HPVEC samples. **Figure S2.** Unsupervised hierarchical clustering of probes showing variable DNA methylation levels (coefficient of variation >0.4) in endothelial cells derived from different tissue compartments. **Figure S3.** Correlation between the HM450 and Sequenom EpiTYPER. Infinium HumanMethylation450 methylation accurately reflects DNA methylation levels in HPAEC and HPVEC. **Figure S4.** Coordinated gene expression and DNA methylation in “Cardiovascular System Development and Function, Connective Tissue Development and Function, Skeletal and Muscular System Development and Function” pathway genes in HPAEC and HPVEC. **Figure S5.** Scatterplot showing relationship between DNA methylation and gene expression in venous and arterial cells. **Figure S6.** Proportion of probes associated with specific gene expression change by genomic location. **Figure S7.** NOS3 Sequenom Assays used to measure regional methylation in HPVEC and HPAEC for Assay 1 (A), and Assay 2 (B). Click here for file

Additional file 2: Table S2 All probes showing higher average β values by 10% or more in HPVEC than HPAEC. Click here for file

Additional file 3: Table S3 All probes showing higher average β values by 10% or more in HPAEC HPVEC. Click here for file

Additional file 4: Table S1 DNMT-associated DNA methylation values in HPAEC and HPVEC cells. Click here for file

Additional file 5: Table S4 IPA (Ingenuity Pathway Analysis) in differentially methylated probes by 10% (DNA methylation) and differentially expressed by 0.5 Log FC (located near transcription start sites). **Table S5.** Gene Ontology analysis in differentially methylated probes by 10% (DNA methylation) and differentially expressed by 0.5 Log FC (located near transcription start sites). **Table S6.** List of probes showing coordinated methylation (less methylated) and gene expression (higher expression) in HPAEC. **Table S7.** List of probes showing coordinated methylation (less methylated) and gene expression (higher expression) in HPVEC. **Table S8.** List of selected probes associated in genes shown in Table 1. **Table S9.** List of probes on selected miRs. Click here for file

## References

[B1] Barker DJ Gluckman PD Godfrey KM Harding JE Owens JA Robinson JS Fetal nutrition and cardiovascular disease in adult life Lancet 1993 341 8850 938 941 10.1016/0140-6736(93)91224-A 8096277

[B2] Harding JE The nutritional basis of the fetal origins of adult disease Int J Epidemiol 2001 30 1 15 23 10.1093/ije/30.1.15 11171842

[B3] Hanson M Gluckman P Endothelial dysfunction and cardiovascular disease: the role of predictive adaptive responses Heart 2005 91 7 864 866 10.1136/hrt.2004.047381 15958346 PMC1769000

[B4] Aitsebaomo J Portbury AL Schisler JC Patterson C Brothers and sisters: molecular insights into arterial-venous heterogeneity Circ Res 2008 103 9 929 939 10.1161/CIRCRESAHA.108.184937 18948631 PMC2760069

[B5] Lawson ND Scheer N Pham VN Kim CH Chitnis AB Campos-Ortega JA Weinstein BM Notch signaling is required for arterial-venous differentiation during embryonic vascular development Development 2001 128 19 3675 3683 11585794 10.1242/dev.128.19.3675

[B6] Torres-Vazquez J Kamei M Weinstein BM Molecular distinction between arteries and veins Cell Tissue Res 2003 314 1 43 59 10.1007/s00441-003-0771-8 14505031

[B7] Swift MR Weinstein BM Arterial-venous specification during development Circ Res 2009 104 5 576 588 10.1161/CIRCRESAHA.108.188805 19286613

[B8] Kume T Specification of arterial, venous, and lymphatic endothelial cells during embryonic development Histol Histopathol 2010 25 5 637 646 20238301 10.14670/hh-25.637 PMC2899674

[B9] Lang I Schweizer A Hiden U Ghaffari-Tabrizi N Hagendorfer G Bilban M Pabst MA Korgun ET Dohr G Desoye G Human fetal placental endothelial cells have a mature arterial and a juvenile venous phenotype with adipogenic and osteogenic differentiation potential Differentiation 2008 76 10 1031 1043 10.1111/j.1432-0436.2008.00302.x 18673379

[B10] Ho M Yang E Matcuk G Deng D Sampas N Tsalenko A Tabibiazar R Zhang Y Chen M Talbi S Identification of endothelial cell genes by combined database mining and microarray analysis Physiol Genomics 2003 13 3 249 262 12644598 10.1152/physiolgenomics.00186.2002

[B11] Fish JE Marsden PA Endothelial nitric oxide synthase: insight into cell-specific gene regulation in the vascular endothelium Cell Mol Life Sci 2006 63 2 144 162 10.1007/s00018-005-5421-8 16416260 PMC11136399

[B12] Matouk CC Marsden PA Epigenetic regulation of vascular endothelial gene expression Circ Res 2008 102 8 873 887 10.1161/CIRCRESAHA.107.171025 18436802

[B13] Napoli C Hayashi T Cacciatore F Casamassimi A Casini C Al-Omran M Ignarro LJ Endothelial progenitor cells as therapeutic agents in the microcirculation: an update Atherosclerosis 2011 215 1 9 22 10.1016/j.atherosclerosis.2010.10.039 21126740

[B14] Bennett-Baker PE Wilkowski J Burke DT Age-associated activation of epigenetically repressed genes in the mouse Genetics 2003 165 4 2055 2062 14704185 10.1093/genetics/165.4.2055 PMC1462878

[B15] Chan Y Fish JE D'Abreo C Lin S Robb GB Teichert AM Karantzoulis-Fegaras F Keightley A Steer BM Marsden PA The cell-specific expression of endothelial nitric-oxide synthase: a role for DNA methylation J Biol Chem 2004 279 33 35087 35100 10.1074/jbc.M405063200 15180995

[B16] Yan MS Matouk CC Marsden PA Epigenetics of the vascular endothelium J Appl Physiol 2010 109 3 916 926 10.1152/japplphysiol.00131.2010 20413423

[B17] Peng Y Jahroudi N The NFY transcription factor inhibits von Willebrand factor promoter activation in non-endothelial cells through recruitment of histone deacetylases J Biol Chem 2003 278 10 8385 8394 10.1074/jbc.M213156200 12511565

[B18] Wu J Iwata F Grass JA Osborne CS Elnitski L Fraser P Ohneda O Yamamoto M Bresnick EH Molecular determinants of NOTCH4 transcription in vascular endothelium Mol Cell Biol 2005 25 4 1458 1474 10.1128/MCB.25.4.1458-1474.2005 15684396 PMC548019

[B19] Johnson AB Barton MC Hypoxia-induced and stress-specific changes in chromatin structure and function Mutat Res 2007 618 1–2 149 162 17292925 10.1016/j.mrfmmm.2006.10.007 PMC1924842

[B20] Fish JE Yan MS Matouk CC St Bernard R Ho JJ Gavryushova A Srivastava D Marsden PA Hypoxic repression of endothelial nitric-oxide synthase transcription is coupled with eviction of promoter histones J Biol Chem 2010 285 2 810 826 10.1074/jbc.M109.067868 19880524 PMC2801283

[B21] Bronneke S Bruckner B Peters N Bosch TC Stab F Wenck H Hagemann S Winnefeld M DNA methylation regulates lineage-specifying genes in primary lymphatic and blood endothelial cells Angiogenesis 2012 15 2 317 329 10.1007/s10456-012-9264-2 22434260

[B22] Palinski W Maternal-fetal cholesterol transport in the placenta: good, bad, and target for modulation Circ Res 2009 104 5 569 571 10.1161/CIRCRESAHA.109.194191 19286612

[B23] Stefulj J Panzenboeck U Becker T Hirschmugl B Schweinzer C Lang I Marsche G Sadjak A Lang U Desoye G Human endothelial cells of the placental barrier efficiently deliver cholesterol to the fetal circulation via ABCA1 and ABCG1 Circ Res 2009 104 5 600 608 10.1161/CIRCRESAHA.108.185066 19168441

[B24] Scholler M Wadsack C Metso J Chirackal Manavalan AP Sreckovic I Schweinzer C Hiden U Jauhiainen M Desoye G Panzenboeck U Phospholipid transfer protein is differentially expressed in human arterial and venous placental endothelial cells and enhances cholesterol efflux to fetal HDL J Clin Endocrinol Metab 2012 97 7 2466 2474 10.1210/jc.2011-2969 22492872

[B25] Novakovic B Yuen RK Gordon L Penaherrera MS Sharkey A Moffett A Craig JM Robinson WP Saffery R Evidence for widespread changes in promoter methylation profile in human placenta in response to increasing gestational age and environmental/stochastic factors BMC Genomics 2011 12 529 10.1186/1471-2164-12-529 22032438 PMC3216976

[B26] Martino D Maksimovic J Joo JH Prescott SL Saffery R Genome-scale profiling reveals a subset of genes regulated by DNA methylation that program somatic T-cell phenotypes in humans Genes Immun 2012 13 5 388 398 10.1038/gene.2012.7 22495533

[B27] Roessler J Ammerpohl O Gutwein J Hasemeier B Anwar SL Kreipe HH Lehmann U Quantitative cross-validation and content analysis of the 450k DNA methylation array from Illumina Inc. BMC Res Notes 2012 5 1 210 10.1186/1756-0500-5-210 PMC3420245 22546179

[B28] Bibikova M Barnes B Tsan C Ho V Klotzle B Le JM Delano D Zhang L Schroth GP Gunderson KL High density DNA methylation array with single CpG site resolution Genomics 2011 98 4 288 295 10.1016/j.ygeno.2011.07.007 21839163

[B29] Dedeurwaerder S Defrance M Calonne E Denis H Sotiriou C Fuks F Evaluation of the Infinium Methylation 450K technology Epigenomics 2011 3 6 771 784 10.2217/epi.11.105 22126295

[B30] Wilcox JN Subramanian RR Sundell CL Tracey WR Pollock JS Harrison DG Marsden PA Expression of multiple isoforms of nitric oxide synthase in normal and atherosclerotic vessels Arterioscler Thromb Vasc Biol 1997 17 11 2479 2488 10.1161/01.ATV.17.11.2479 9409218

[B31] Fish JE Matouk CC Rachlis A Lin S Tai SC D'Abreo C Marsden PA The expression of endothelial nitric-oxide synthase is controlled by a cell-specific histone code J Biol Chem 2005 280 26 24824 24838 10.1074/jbc.M502115200 15870070

[B32] Gale NW Yancopoulos GD Growth factors acting via endothelial cell-specific receptor tyrosine kinases: VEGFs, angiopoietins, and ephrins in vascular development Genes Dev 1999 13 9 1055 1066 10.1101/gad.13.9.1055 10323857

[B33] Edelstein LC Pan A Collins T Chromatin modification and the endothelial-specific activation of the E-selectin gene J Biol Chem 2005 280 12 11192 11202 10.1074/jbc.M412997200 15671023 PMC1382061

[B34] Morishita K Johnson DE Williams LT A novel promoter for vascular endothelial growth factor receptor (flt-1) that confers endothelial-specific gene expression J Biol Chem 1995 270 46 27948 27953 10.1074/jbc.270.46.27948 7499271

[B35] Eichmann A Yuan L Moyon D Lenoble F Pardanaud L Breant C Vascular development: from precursor cells to branched arterial and venous networks Int J Dev Biol 2005 49 2–3 259 267 15906240 10.1387/ijdb.041941ae

[B36] Jjingo D Conley AB Yi SV Lunyak VV Jordan IK On the presence and role of human gene-body DNA methylation Oncotarget 2012 3 4 462 474 22577155 10.18632/oncotarget.497 PMC3380580

[B37] Aran D Toperoff G Rosenberg M Hellman A Replication timing-related and gene body-specific methylation of active human genes Hum Mol Genet 2011 20 4 670 680 10.1093/hmg/ddq513 21112978

[B38] Ball MP Li JB Gao Y Lee JH LeProust EM Park IH Xie B Daley GQ Church GM Targeted and genome-scale strategies reveal gene-body methylation signatures in human cells Nat Biotechnol 2009 27 4 361 368 10.1038/nbt.1533 19329998 PMC3566772

[B39] Tsutsui M Shimokawa H Otsuji Y Yanagihara N Pathophysiological relevance of NO signaling in the cardiovascular system: novel insight from mice lacking all NO synthases Pharmacol Ther 2010 128 3 499 508 10.1016/j.pharmthera.2010.08.010 20826180

[B40] Masood R Cai J Zheng T Smith DL Hinton DR Gill PS Vascular endothelial growth factor (VEGF) is an autocrine growth factor for VEGF receptor-positive human tumors Blood 2001 98 6 1904 1913 10.1182/blood.V98.6.1904 11535528

[B41] Kim JY Hwang JH Zhou W Shin J Noh SM Song IS Kim JY Lee SH Kim J The expression of VEGF receptor genes is concurrently influenced by epigenetic gene silencing of the genes and VEGF activation Epigenetics 2009 4 5 313 321 10.4161/epi.4.5.9160 19633424

[B42] Shibuya M Structure and dual function of vascular endothelial growth factor receptor-1 (Flt-1) Int J Biochem Cell Biol 2001 33 4 409 420 10.1016/S1357-2725(01)00026-7 11312109

[B43] Ferrer FA Miller LJ Lindquist R Kowalczyk P Laudone VP Albertsen PC Kreutzer DL Expression of vascular endothelial growth factor receptors in human prostate cancer Urology 1999 54 3 567 572 10.1016/S0090-4295(99)00156-9 10475375

[B44] Veikkola T Karkkainen M Claesson-Welsh L Alitalo K Regulation of angiogenesis via vascular endothelial growth factor receptors Cancer Res 2000 60 2 203 212 10667560

[B45] Quentmeier H Eberth S Romani J Weich HA Zaborski M Drexler HG DNA methylation regulates expression of VEGF-R2 (KDR) and VEGF-R3 (FLT4) BMC Cancer 2012 12 19 10.1186/1471-2407-12-19 22251800 PMC3297533

[B46] Fitzner N Clauberg S Essmann F Liebmann J Kolb-Bachofen V Human skin endothelial cells can express all 10 TLR genes and respond to respective ligands Clinical and vaccine immunology: CVI 2008 15 1 138 146 10.1128/CVI.00257-07 17978010 PMC2223852

[B47] Diesel B Ripoche N Risch RT Tierling S Walter J Kiemer AK Inflammation-induced up-regulation of TLR2 expression in human endothelial cells is independent of differential methylation in the TLR2 promoter CpG island Innate Immun 2012 18 1 112 123 10.1177/1753425910394888 21768203

[B48] Wu F Yang Z Li G Role of specific microRNAs for endothelial function and angiogenesis Biochem Biophys Res Commun 2009 386 4 549 553 10.1016/j.bbrc.2009.06.075 19540203 PMC2821898

[B49] Choi YC Yoon S Jeong Y Yoon J Baek K Regulation of vascular endothelial growth factor signaling by miR-200b Mol Cells 2011 32 1 77 82 10.1007/s10059-011-1042-2 21544626 PMC3887663

[B50] Fang Y Shi C Manduchi E Civelek M Davies PF MicroRNA-10a regulation of proinflammatory phenotype in athero-susceptible endothelium in vivo and in vitro Proc Natl Acad Sci USA 2010 107 30 13450 13455 10.1073/pnas.1002120107 20624982 PMC2922125

[B51] Muramatsu F Kidoya H Naito H Sakimoto S Takakura N MicroRNA-125b inhibits tube formation of blood vessels through translational suppression of VE-cadherin Oncogene 2012 32 4 414 421 22391569 10.1038/onc.2012.68

[B52] da Huang W Sherman BT Lempicki RA Systematic and integrative analysis of large gene lists using DAVID bioinformatics resources Nat Protoc 2009 4 1 44 57 19131956 10.1038/nprot.2008.211

[B53] Lassance L Miedl H Konya V Heinemann A Ebner B Hackl H Desoye G Hiden U Differential response of arterial and venous endothelial cells to extracellular matrix is modulated by oxygen Histochem Cell Biol 2012 Epub 10.1007/s00418-012-0917-4 22294260

[B54] Novakovic B Wong NC Sibson M Ng HK Morley R Manuelpillai U Down T Rakyan VK Beck S Hiendleder S DNA methylation-mediated down-regulation of DNA methyltransferase-1 (DNMT1) is coincident with, but not essential for, global hypomethylation in human placenta J Biol Chem 2010 285 13 9583 9593 10.1074/jbc.M109.064956 20071334 PMC2843208

[B55] Gordon L Joo JE Powell JE Ollikainen M Novakovic B Li X Andronikos R Cruickshank MN Conneely KN Smith AK Neonatal DNA methylation profile in human twins is specified by a complex interplay between intrauterine environmental and genetic factors, subject to tissue-specific influence Genome Res 2012 22 8 1395 1406 10.1101/gr.136598.111 22800725 PMC3409253

[B56] Hansen KD Aryee M Minfi: Analyze Illumina's 450k methylation arrays R package version 100

[B57] Maksimovic J Gordon L Oshlack A SWAN: Subset quantile Within-Array Normalization for Illumina Infinium Human Methylation450 BeadChips Genome Biol 2012 13 6 R44 10.1186/gb-2012-13-6-r44 22703947 PMC3446316

[B58] Reiner A Yekutieli D Benjamini Y Identifying differentially expressed genes using false discovery rate controlling procedures Bioinformatics 2003 19 3 368 375 10.1093/bioinformatics/btf877 12584122

[B59] Du P Kibbe WA Lin SM lumi: a pipeline for processing Illumina microarray Bioinformatics (Oxford, England) 2008 24 13 1547 1548 10.1093/bioinformatics/btn224 18467348

[B60] Smyth GK Gentleman R, Carey V, Dudoit S, Huber W, Irizarry R **Limma: linear models for microarray data**. In: *Bioinformatics and Computational Biology Solutions using R and Bioconducto* 2005 New York: Springer 397 420

[B61] da Huang W Sherman BT Lempicki RA Bioinformatics enrichment tools: paths toward the comprehensive functional analysis of large gene lists Nucleic Acids Res 2009 37 1 1 13 10.1093/nar/gkn923 19033363 PMC2615629

[B62] Ollikainen M Smith KR Joo EJ Ng HK Andronikos R Novakovic B Abdul Aziz NK Carlin JB Morley R Saffery R DNA methylation analysis of multiple tissues from newborn twins reveals both genetic and intrauterine components to variation in the human neonatal epigenome Hum Mol Genet 2010 19 21 4176 4188 10.1093/hmg/ddq336 20699328

[B63] Wong NC Novakovic B Weinrich B Dewi C Andronikos R Sibson M Macrae F Morley R Pertile MD Craig JM Methylation of the adenomatous polyposis coli (APC) gene in human placenta and hypermethylation in choriocarcinoma cells Cancer Lett 2008 268 1 56 62 10.1016/j.canlet.2008.03.033 18485586

