# Supplementary material for: Variable promoter methylation contributes to differential expression of key genes in human placenta-derived venous and arterial endothelial cells
Source: BMC Genomics. 2013 Jul 15;14:475. doi: 10.1186/1471-2164-14-475 (PMC3729658; doi:10.1186/1471-2164-14-475)
Supplement: Additional file 1: Figure S1 — Unsupervised hierarchical clustering of probes showing variable DNA methylation levels (coefficient of variation >0.4) in HPAEC and HPVEC samples. Figure S2. Unsupervised hierarchical clustering of probes showing variable DNA methylation levels (coefficient of variation >0.4) in endothelial cells derived from different tissue compartments. Figure S3. Correlation between the HM450 and Sequenom EpiTYPER. Infinium HumanMethylation450 methylation accurately reflects DNA methylation levels in HPAEC and HPVEC. Figure S4. Coordinated gene expression and DNA methylation in “Cardiovascular System Development and Function, Connective Tissue Development and Function, Skeletal and Muscular System Development and Function” pathway genes in HPAEC and HPVEC. Figure S5. Scatterplot showing relationship between DNA methylation and gene expression in venous and arterial cells. Figure S6. Proportion of probes associated with specific gene expression change by genomic location. Figure S7. NOS3 Sequenom Assays used to measure regional methylation in HPVEC and HPAEC for Assay 1 (A), and Assay 2 (B). [file 1471-2164-14-475-S1.ppt]

## Slide 1
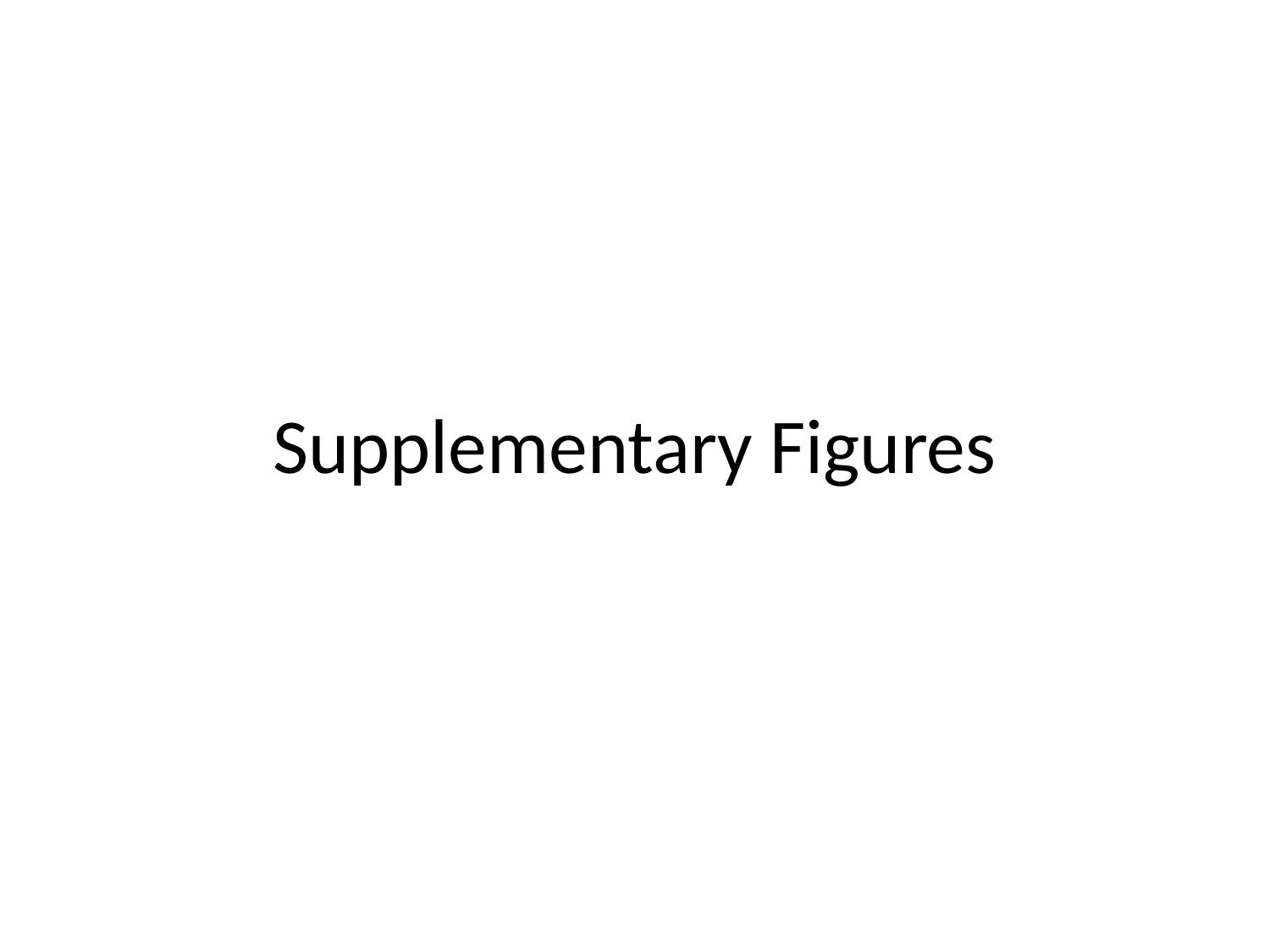

# Supplementary Figures

## Slide 2
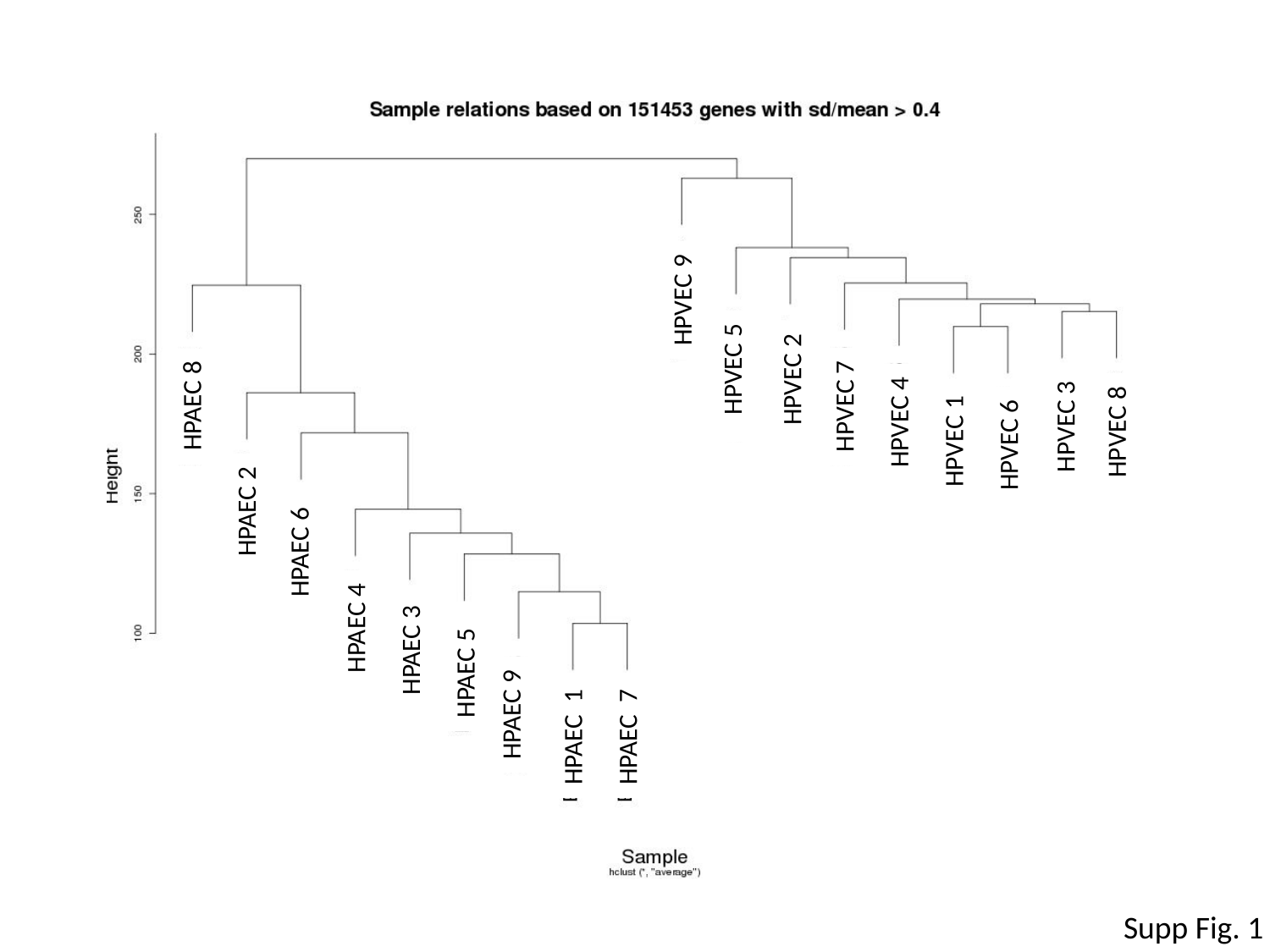

HPVEC 9
HPVEC 5
HPVEC 2
HPAEC 8
HPVEC 7
HPVEC 4
HPVEC 3
HPVEC 8
HPVEC 1
HPVEC 6
HPAEC 2
HPAEC 6
HPAEC 4
HPAEC 3
HPAEC 5
HPAEC 9
HPAEC 1
HPAEC 7
Supp Fig. 1

## Slide 3
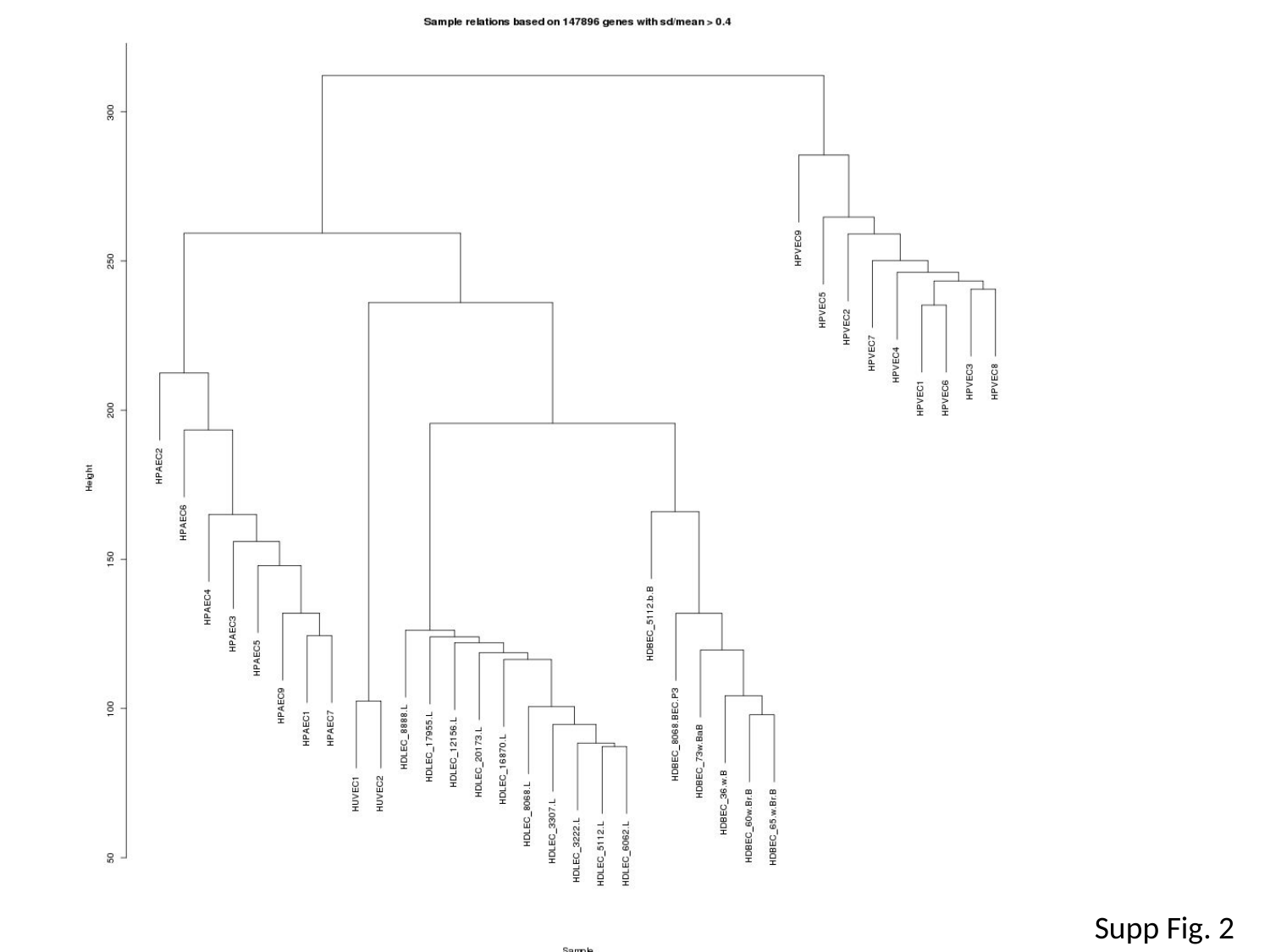

Supp Fig. 2

## Slide 4
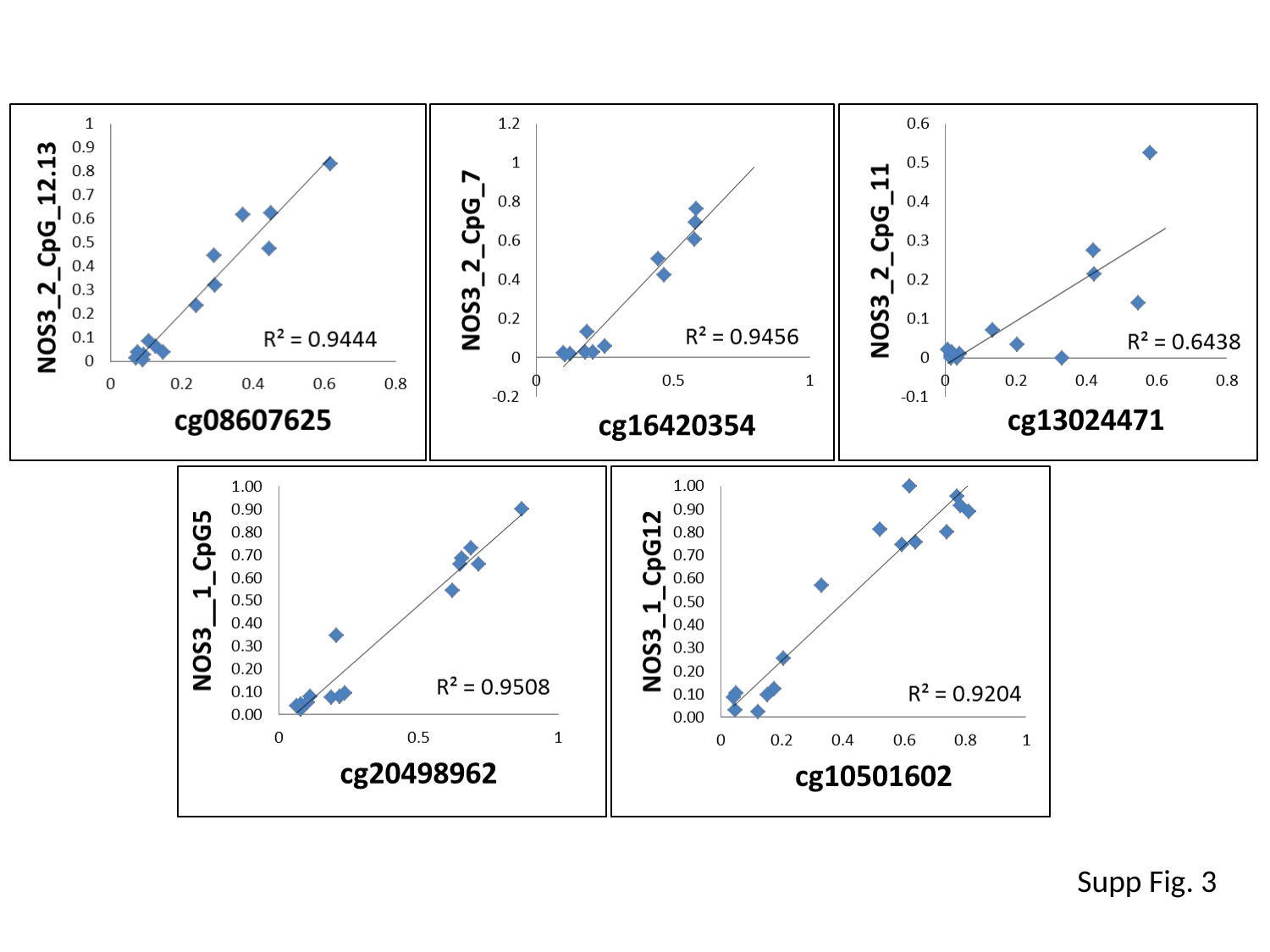

Supp Fig. 3

## Slide 5
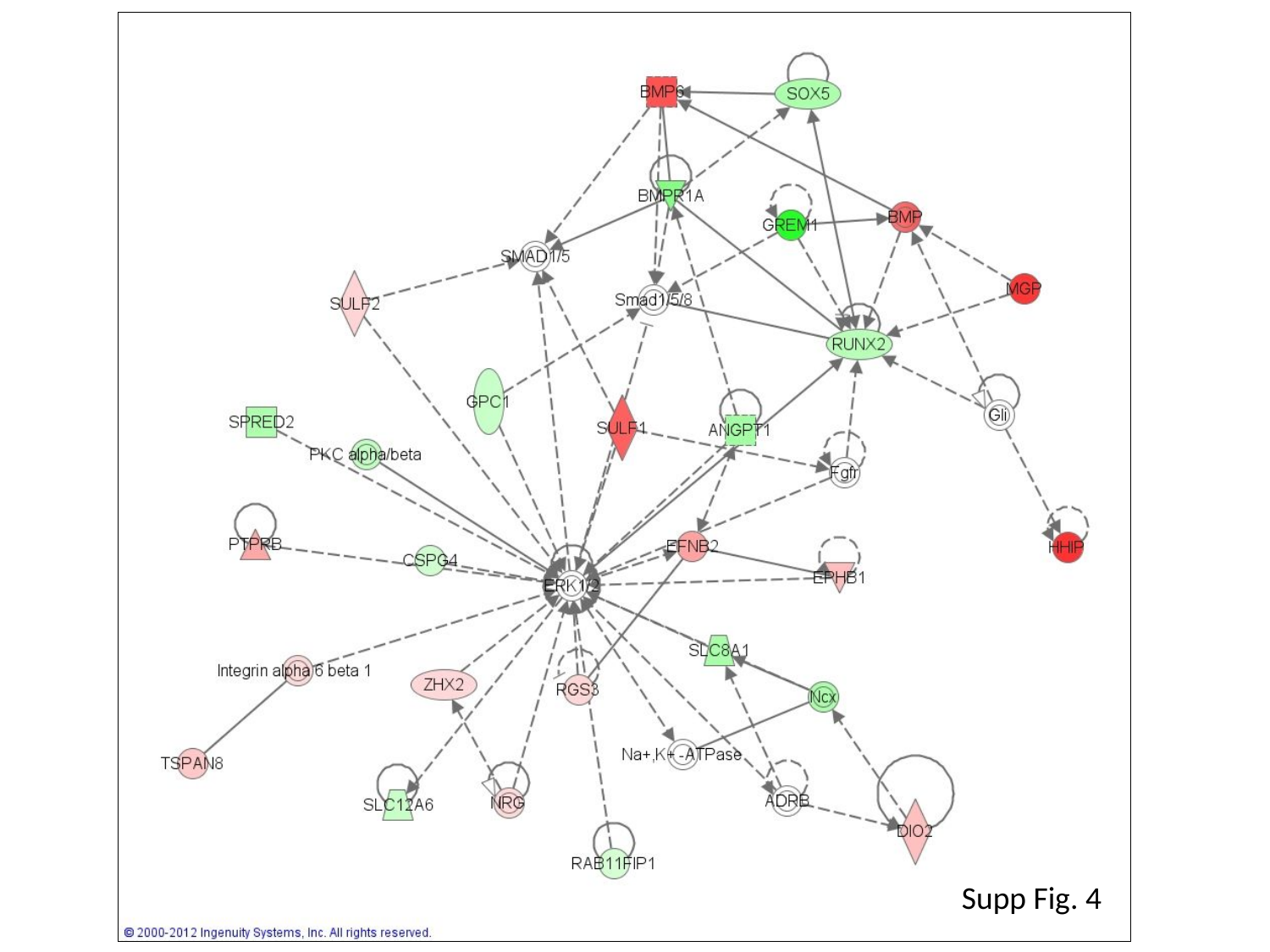

Supp Fig. 4

## Slide 6
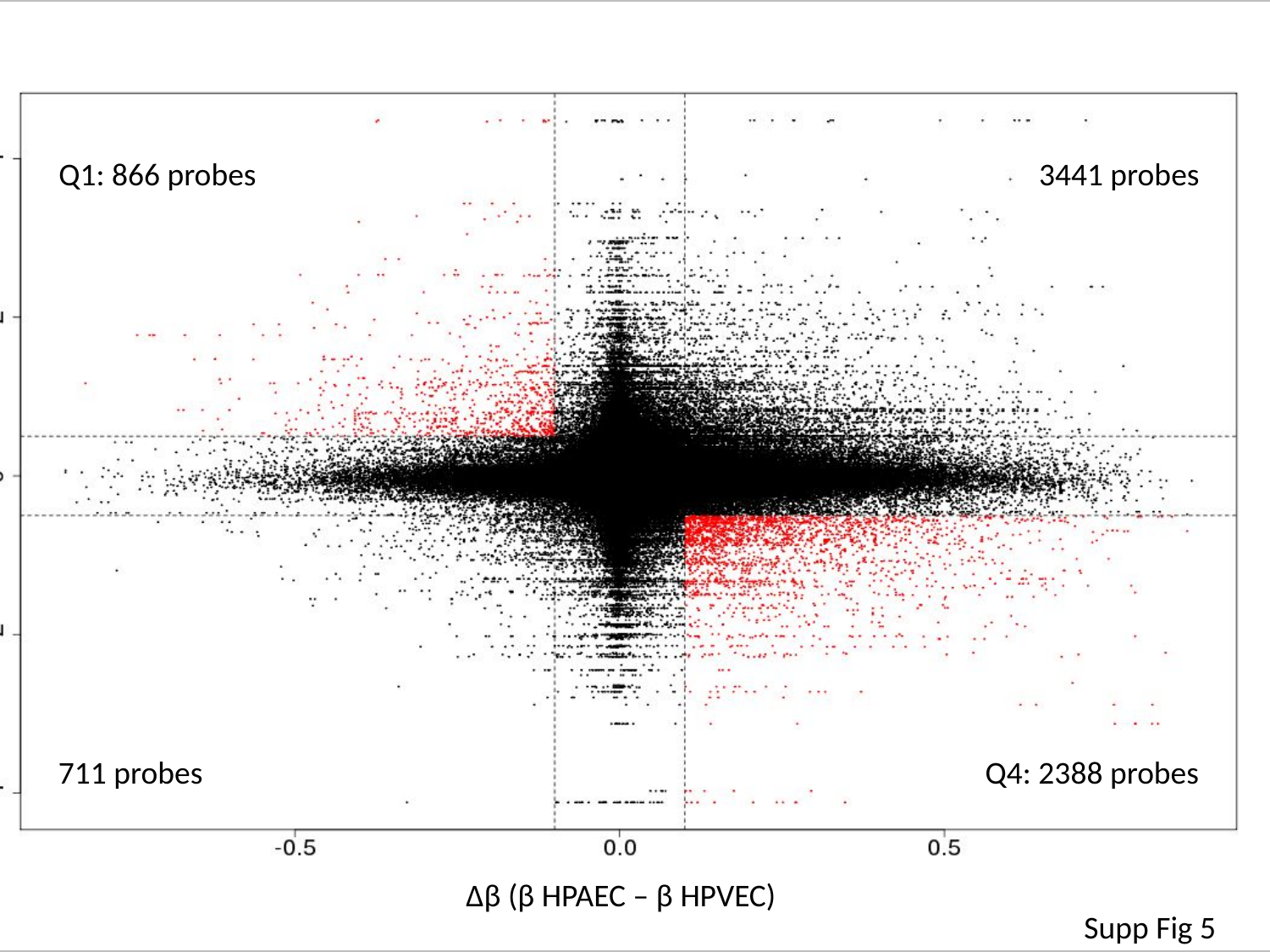

Q1: 866 probes
3441 probes
Log Fold Change
Expression Difference (HPAEC – HPVEC)
711 probes
Q4: 2388 probes
Δβ (β HPAEC – β HPVEC)
Supp Fig 5

## Slide 7
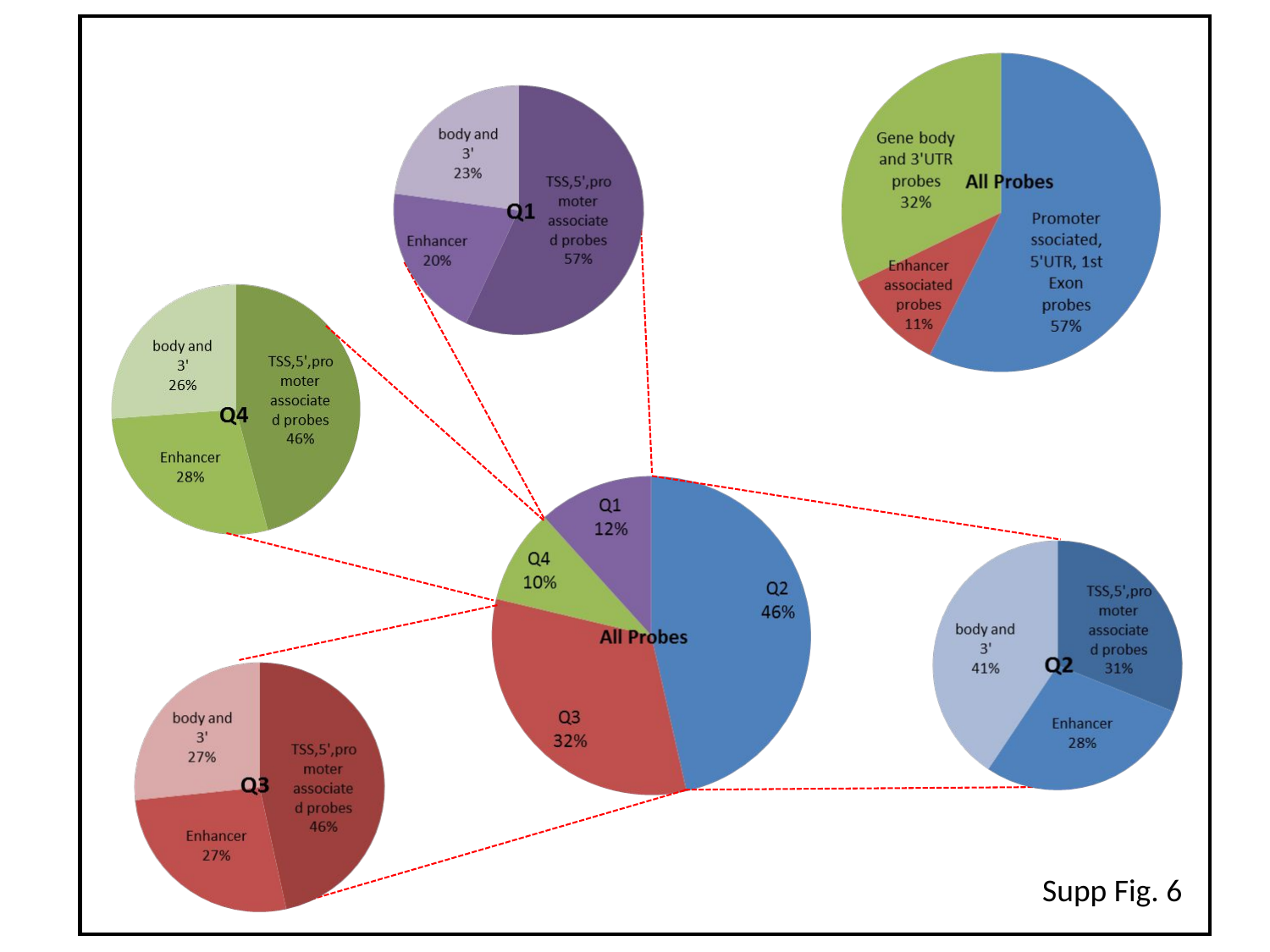

Supp Fig. 6

## Slide 8
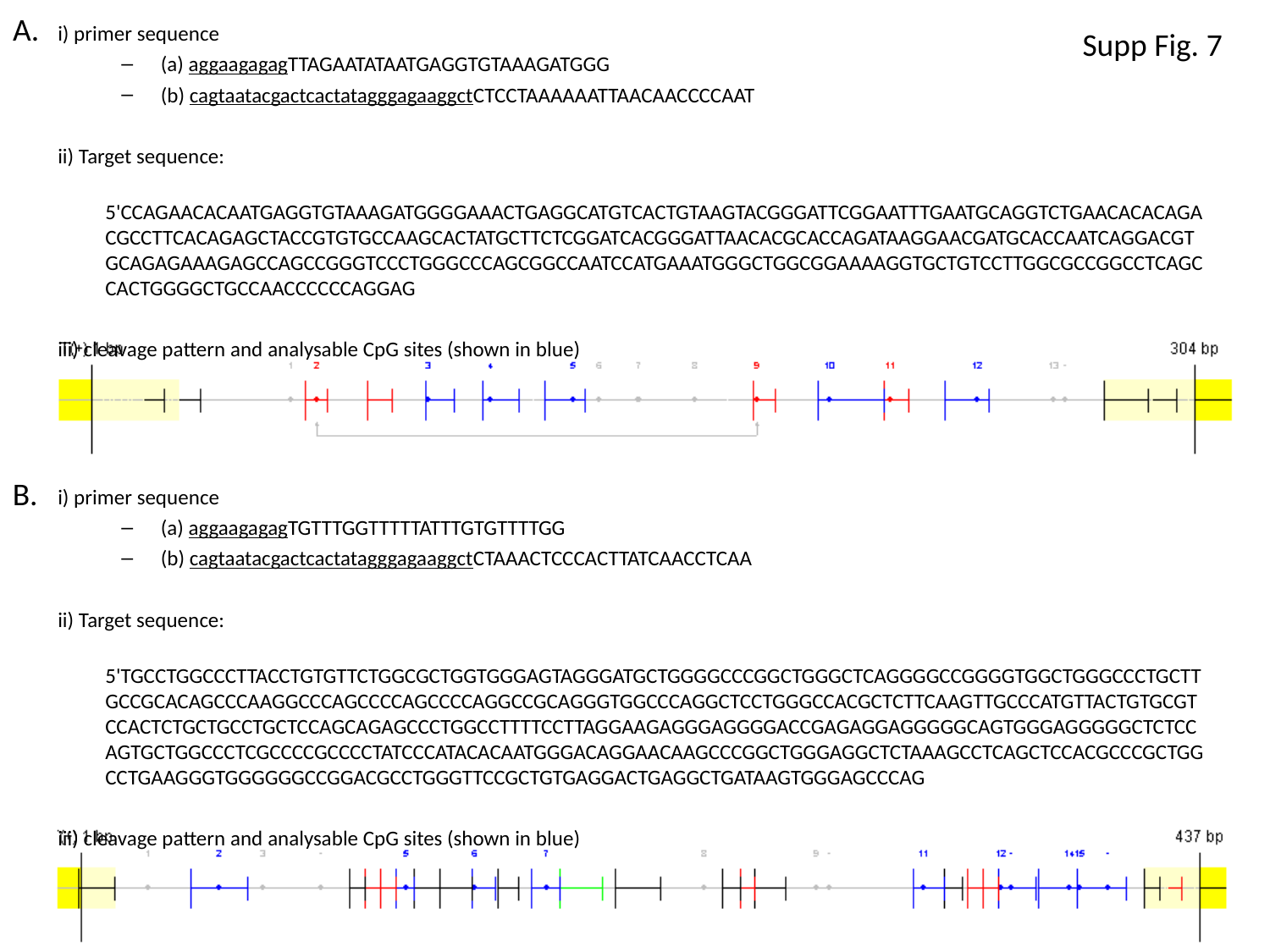

A.
i) primer sequence
(a) aggaagagagTTAGAATATAATGAGGTGTAAAGATGGG
(b) cagtaatacgactcactatagggagaaggctCTCCTAAAAAATTAACAACCCCAAT
ii) Target sequence:
	5'CCAGAACACAATGAGGTGTAAAGATGGGGAAACTGAGGCATGTCACTGTAAGTACGGGATTCGGAATTTGAATGCAGGTCTGAACACACAGACGCCTTCACAGAGCTACCGTGTGCCAAGCACTATGCTTCTCGGATCACGGGATTAACACGCACCAGATAAGGAACGATGCACCAATCAGGACGTGCAGAGAAAGAGCCAGCCGGGTCCCTGGGCCCAGCGGCCAATCCATGAAATGGGCTGGCGGAAAAGGTGCTGTCCTTGGCGCCGGCCTCAGCCACTGGGGCTGCCAACCCCCCAGGAG
iii) cleavage pattern and analysable CpG sites (shown in blue)
Supp Fig. 7
B.
i) primer sequence
(a) aggaagagagTGTTTGGTTTTTATTTGTGTTTTGG
(b) cagtaatacgactcactatagggagaaggctCTAAACTCCCACTTATCAACCTCAA
ii) Target sequence:
	5'TGCCTGGCCCTTACCTGTGTTCTGGCGCTGGTGGGAGTAGGGATGCTGGGGCCCGGCTGGGCTCAGGGGCCGGGGTGGCTGGGCCCTGCTTGCCGCACAGCCCAAGGCCCAGCCCCAGCCCCAGGCCGCAGGGTGGCCCAGGCTCCTGGGCCACGCTCTTCAAGTTGCCCATGTTACTGTGCGTCCACTCTGCTGCCTGCTCCAGCAGAGCCCTGGCCTTTTCCTTAGGAAGAGGGAGGGGACCGAGAGGAGGGGGCAGTGGGAGGGGGCTCTCCAGTGCTGGCCCTCGCCCCGCCCCTATCCCATACACAATGGGACAGGAACAAGCCCGGCTGGGAGGCTCTAAAGCCTCAGCTCCACGCCCGCTGGCCTGAAGGGTGGGGGGCCGGACGCCTGGGTTCCGCTGTGAGGACTGAGGCTGATAAGTGGGAGCCCAG
iii) cleavage pattern and analysable CpG sites (shown in blue)
